# A sensitization strategy for highly efficient blue fluorescent organic light-emitting diodes

**DOI:** 10.1007/s12200-022-00046-z

**Published:** 2022-11-10

**Authors:** Yalei Duan, Runda Guo, Yaxiong Wang, Kaiyuan Di, Lei Wang

**Affiliations:** Wuhan National Laboratory for Optoelectronics, Huazhong University of Science and Technology, Wuhan, 430074 China

**Keywords:** Organic light-emitting diodes, Hyperfluorescence, Pyrene, Sensitization strategy, Blue fluorescent

## Abstract

Highly efficient blue fluorescent materials have recently attracted great interest for organic light-emitting diode (OLED) application. Here, two new pyrene based organic molecules consisting of a highly rigid skeleton, namely **SPy** and **DPy**, are developed. These two blue light emitters exhibit excellent thermal stability. The experiment reveals that the full-width at half-maximum (FWHM) of the emission spectrum can be tuned by introducing different amounts of 9,9-diphenyl-*N*-phenyl-9H-fluoren-2-amine on pyrene units. The FWHM of the emission spectrum is only 37 nm in diluted toluene solution for **DPy**. Furthermore, highly efficient blue OLEDs are obtained by thermally activated delayed fluorescence (TADF) sensitization strategy. The blue fluorescent OLEDs utilizing **DPy** as emitters achieve a maximum external quantum efficiency (EQE) of 10.4% with the electroluminescence (EL) peak/FWHM of 480 nm/49 nm. Particularly, the EQE of **DPy**-based device is boosted from 2.6% in non-doped device to 10.4% in DMAc-DPS TADF sensitized fluorescence (TSF) device, which is a 400% enhancement. Therefore, this work demonstrates that the TSF strategy is promising for highly efficient fluorescent OLEDs application in wide-color-gamut display field.

**Graphical Abstract:**

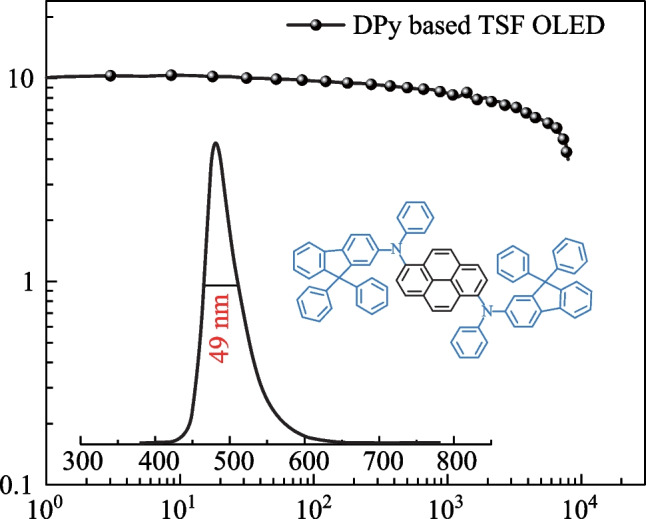

**Supplementary Information:**

The online version contains supplementary material available at 10.1007/s12200-022-00046-z.

## Introduction

Organic light-emitting diodes (OLEDs) have attracted enormous attention in the last decade and play an important role in new-generation flat-panel displays and solid-state lighting [1–13]. According to the spin statistics under electrical excitation, the recombination of holes and electrons injected into OLED active layers is expected to produce singlet and triplet excitons in a 25%:75% (1:3) ratio [10, 14–16]. Unfortunately, only the 25% singlet excitons are luminescent in fluorescent organic light-emitting diodes (FOLEDs) [17]. In contrast, thermally activated delayed fluorescence (TADF) emitters can obtain internal quantum efficiency (IQE) of nearly 100% via reverse intersystem crossing (RISC) based on an efficient triplet-to-singlet process, and has emerged as a promising alternative to the more traditional phosphorescence and conventional fluorescence [16, 18, 19]. However, the TADF emitters generally have a broad emission spectrum with a large FWHM, typically up to 70–100 nm, primarily owing to structural relaxations in the excited state and distinct intramolecular charge transfer (ICT) nature of the emissive S_1_ states [20–25]. Such a broadening of the emission spectrum greatly affects color purity of OLED devices [26].

Recently, several approaches to obtain high IQE in fluorescent molecules and high external quantum efficiency (EQE) of electroluminescence (EL) have been put forward. An exceptional solution for this problem is “hyperfluorescence”, which is aimed at obtaining OLED emitters with high EQE and good color purity simutaneously [27]. Typically, the TADF, exhibiting IQEs of 100%, has been chosen as a sensitizer in a three-component emissive layer [28]. With such a system, the fluorescent emitters can obtian an IQE higher than 25%, due to the Förster resonance energy transfer (FRET) taking place from the excited singlet state of the TADF molecule to the excited singlet state of the fluorescent molecule [29]. The fluorophores that exhibit narrow emission spectra can improve color purity and also obtain high EQE in hyperfluorescence. On the other hand, pyrene, a member of polycyclic aromatic hydrocarbon (PAH) class of material, has attracted considerable attention because of its good luminescent properties [30–33]. Some pyrene derivatives decorated by specific groups have exihibited narrowband emission, and provide the possibility to explore conventional narrowband fluorescent materials [34–37]. For example, Kang et al. developed a blue emitter 2CN, and achieved an EQE of 4.1% in OLEDs, with FWHM of 46 nm [38]. Later, Kim et al. reported a blue non-doped emitter BDPP with EQE of 3.69% and FWHM of 50 nm [39]. Although those emitters demonstrated excellent FWHM, the device performance was limited by the low maximum internal efficiency (25%) of traditional fluorescence. Interestingly, the emitter could achieve a higher EQE of 13.0% in TADF sensitized fluorescence (TSF) device, as reported by Kwon et al. [34]. Therefore, a sensitization strategy is an effective approach to improve the device efficiency while maintaining narrowband emission.

With the aim of developing fluorescent materials suitable for TSF OLEDs, we designed and synthesized two fluorescent emitters, namely *N*-(9,9-diphenyl-9*H*-fluoren-2-yl)-*N*-phenylpyren-1-amine (**SPy**) and *N*^1^,*N*^6^-bis(9,9-diphenyl-9*H*-fluoren-2-yl)-*N*^1^,*N*^6^-diphenylpyrene-1,6-diamine (**DPy**). With a highly twisted structure and the increase of molecular distance in the film state, the self-quenching of **DPy,** symmetrically embellished by 9,9-diphenyl-*N*-phenyl-9H-fluoren-2-amine, resulted in suppression of a larger spatial highest occupied molecular orbital (HOMO) [40–44]. Furthermore, due to conjugated structure interrupted by the N atom with SP3 hybridized orbital, **DPy** could obtain a short-range charge density shift with narrow band emission. Consequently, **DPy** showed narrowband emission with FWHM value of 37 nm in diluted toluene solution. In particular, when **DPy** was utilized for the fabrication of non-doped OLED, the maximum EQE of the device is only 2.6%, but when adopting a TADF emitter of 10,10′-(sulfonylbis(4,1-phenylene))bis(9,9-dimethyl-9,10-dihydroacridine) (DMAC-DPS) in the TSF system, the maximum EQE could reach a record level of 10.4%, while maintaining a narrowband emission. Therefore, this paper offers a reference strategy to improve the color purity of OLEDs.

## Experimental section

### Materials and reagents

All the solvents and reagents used for target compounds were purchased from commercial suppliers without further purification.

### Synthesis

#### 9, 9-diphenyl-*N*-phenyl-9*H*-fluoren-2-amine (compound D1)

The mixture of 2-bromo-9,9-diphenylfluorene (5.0 g, 12.6 mmol), aniline (1.3 g, 13.8 mmol), Pd_2_(dba)_3_ (0.12 g, 0.13 mmol), P(*t*-Bu)_3_HBF_4_ (0.11 g, 0.38 mmol) and *t*-BuONa (3.6 g, 37.8 mmol) was suspended in toluene (80 mL) and refluxed for 10 h under N_2_ atmosphere at 120 °C. The reaction mixture was cooled to room temperature, and the toluene was removed by vacuum distillation. Then the crude product was purified by column chromatography (elution solvent: petroleum ether) to obtain a white solid (4.7 g), yield, 93%. Nuclear magnetic resonance (NMR) data were as follows: ^1^H NMR (400 MHz, DMSO) *δ* [ppm]: 8.36 (s, 1H), 7.76 (d, *J* = 8.4 Hz, 2H), 7.40–7.16 (m, 11H), 7.12 (dd, *J* = 9.4, 4.5 Hz, 5H), 7.02 (dd, *J* = 12.4, 4.8 Hz, 3H), 6.81 (t, *J* = 7.3 Hz, 1H). Fourier transform high resolution mass spectrometer (FTMS) with Atmospheric Pressure Chemical Ionization (APCI) Source: calculated for C_31_H_23_N, 409.1830; found, 410.1902.

#### *N*-(9,9-diphenyl-9*H*-fluoren-2-yl)-*N*-phenylpyren-1-amine (SPy)

The mixture of 1-bromopyrene (1.45 g, 5.45 mmol), 9,9-diphenyl-N-phenyl-9H-fluoren-2-amine (compound D1) (2.0 g, 4.89 mmol), Pd_2_(dba)_3_ (0.04 g, 0.05 mmol), S-phos (0.06 g, 0.15 mmol) and t-BuONa (1.4 g, 14.7 mmol) was suspended in toluene (50 mL) and refluxed for 16 h under N_2_ atmosphere at 120 °C. The reaction mixture was cooled to room temperature, and the toluene was removed by vacuum distillation. Then the crude product was purified by column chromatography (elution solvent: petroleum ether) to obtain a virescent solid (2.9 g), yield, 96%. ^1^H NMR (400 MHz, DMSO) *δ* [ppm]: 8.36–8.30 (m, 2H), 8.26 (d, *J* = 7.1 Hz, 1H), 8.20 (s, 2H), 8.13–8.04 (m, 2H), 7.99 (d, *J* = 9.3 Hz, 1H), 7.85 (d, *J* = 8.2 Hz, 1H), 7.79 (d, *J* = 8.2 Hz, 2H), 7.36 (dd, *J* = 9.6, 8.0 Hz, 2H), 7.23 (dd, *J* = 10.8, 5.1 Hz, 3H), 7.08 (d, *J* = 5.3 Hz, 6H), 6.99 (dd, *J* = 9.2, 5.8 Hz, 4H), 6.94–6.87 (m, 5H). ^13^C NMR (101 MHz, CDCl_3_) *δ* [ppm]: 152.21, 150.34, 148.15, 148.05, 145.48, 140.48, 139.76, 133.55, 130.72, 128.76, 127.68, 127.37, 127.05, 126.70, 126.09, 125.85, 125.60, 124.74, 122.99, 121.55, 120.43, 119.11, 65.07. FTMS (APCI): calculated for C_47_H_31_N, 609.2457; found, 610.2529, [M]^+^.

#### *N*^1^,*N*^6^-bis(9,9-diphenyl-9*H*-fluoren-2-yl)-*N*^1^,*N*^6^-diphenylpyrene-1,6-diamine (DPy)

The synthetic procedure was similar to that of **SPy**. Yellow solid was obtained (3.5 g), yield, 78%. ^1^H NMR (600 MHz, C_6_D_6_) *δ* [ppm]: 8.15 (d, *J* = 9.2 Hz, 2H), 7.66 (d, *J* = 8.2 Hz, 2H), 7.61 (d, *J* = 8.1 Hz, 2H), 7.52–7.46 (m,7H), 7.44 (d, *J* = 2.0 Hz, 2H), 7.31 (t, *J* = 10.6 Hz, 3H), 7.14 (s, 3H), 7.10 (dd, *J* = 8.3, 2.1 Hz, 3H), 7.05–6.99 (m, 12H), 6.84 (m, *J* = 12.5, 11.7, 5.3 Hz, 16H). ^13^C NMR (151 MHz, C_6_D_6_) *δ* [ppm]: δ 153.36, 151.48, 149.24, 148.94, 146.39, 141.52, 140.58, 134.58, 129.86, 129.57, 128.52, 128.46, 127.18, 126.73, 126.65, 126.43, 123.42, 122.83, 122.45, 121.89, 121.39, 120.40, 119.82. FTMS (APCI): calculated for C_78_H_52_N_2_, 1016.4130; found, 1017.4197, [M]^+^.

## Results and discussion

### Synthesis and characterization

**Scheme 1:**
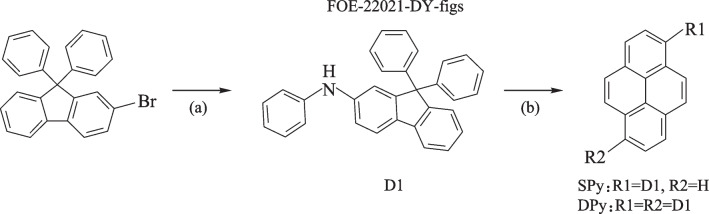
Synthetic route of SPy and DPy: **a** Pd_2_(dba)_3_, P(*t*-Bu)_3_HBF_4_, *t*-BuONa, toluene, 120 °C, 10 h. **b** Pd_2_(dba)_3_, S-phos, *t*-BuONa, toluene, 120 °C, 16 h

The synthetic routes and chemical structures of **SPy** and **DPy** are presented in Scheme 1. The intermediate compound 9,9-diphenyl-*N*-phenyl-9*H*-fluoren-2-amine (**D1)** was synthesized using the Buchwald-Hartwig reaction. **SPy** and **DPy** were obtained via the Buchwald-Hartwig reaction of **D1** and 1-bromopyrene or 1,6-dibromopyrene, respectively [45, 46]. The specific synthesis processes, the related characterization as determined by NMR spectroscopy and mass spectroscopy analysis are given in the experimental section. Before measurements and device fabrication procedure, purification of the target compounds via temperature-gradient sublimation under vacuum conditions was carried out.

### Theoretical calculations

**Fig. 1:**
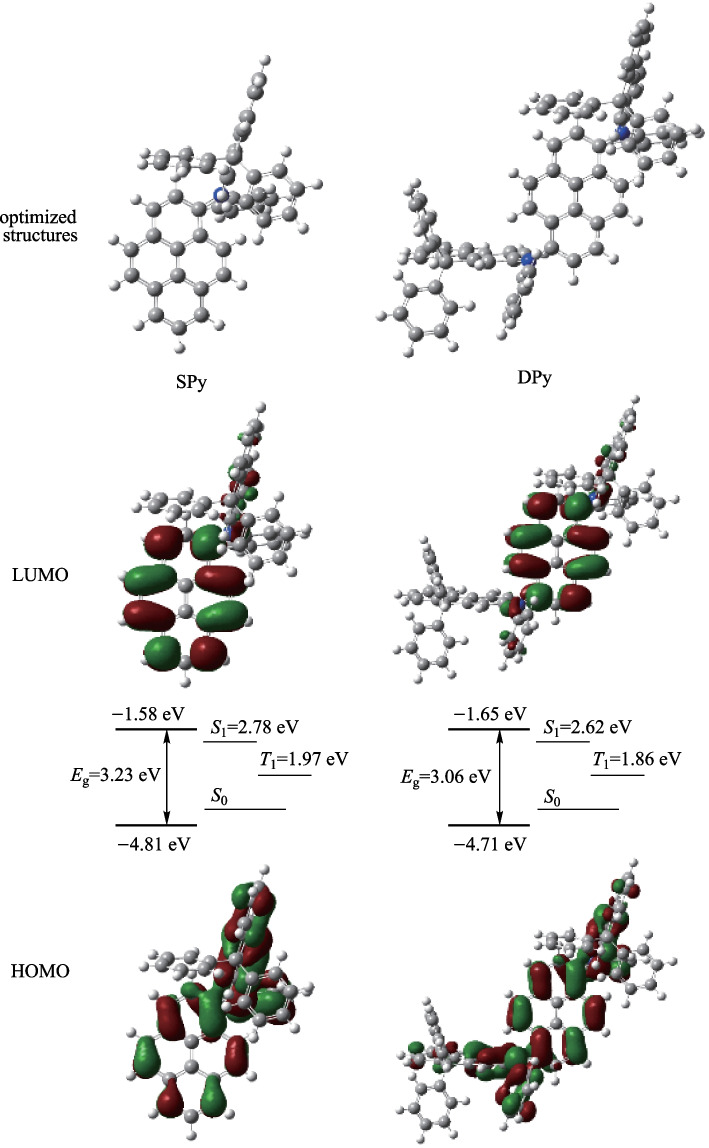
Optimized geometric structures and HOMO/LUMO analysis of **SPy** and **DPy** (the excited state energy levels S_1_/T_1_ are extra labeled)

The frontier orbital distributions of the **SPy** and **DPy** were investigated by using density functional theory (DFT) with B3LYP/6-31G(d) levels to understand the effect of the two materials. The optimized geometries, the frontier molecular orbital (FMO) distributions of the compounds, and the energy band gaps are depicted in Fig. 1. The lowest unoccupied molecular orbitals (LUMO) of these compounds were similar and showed significant contributions to the pyrene units, and distributed on the nitrogen atoms mildly. Meanwhile, the highest occupied molecular orbitals (HOMO) were mainly localized on the pyrene skeleton as well as on the extended the phenyl-rings linked with nitrogen atoms. In contrast, the HOMO and LUMO were not distributed on the phenyl-rings linked with fluorenyl due to the phenyl being separated by the large steric hindrance. The calculated HOMO energies of **SPy** and **DPy** were − 4.81 and − 4.71 eV, respectively. And the energies of LUMO were − 1.58 and − 1.65 eV, respectively. To further explore the excited state properties of these compounds, the S_1_ and T_1_ excited energies were calculated next using time-dependent density functional theory (TD-DFT) with B3LYP/6-31G(d) level based on the ground state geometries presented in Fig. 1. The calculated singlet/triplet energies were 2.78/1.97 eV for **SPy**, and 2.62/1.86 eV for **DPy**, respectively. The large theoretical energy gap between the lowest singlet and triplet states (0.76 eV for DPy and 0.81 eV for SPy) demonstrated that these emitters were not potential TADF materials.

### Thermal and electrochemical properties

**Fig. 2:**
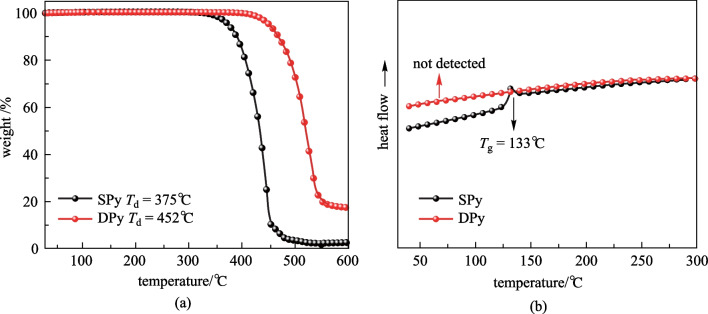
**a** TGA and **b** DSC curves of **SPy** and **DPy**

The thermal stabilities of **SPy** and **DPy** were measured by thermogravimetric analysis (TGA) and differential scanning calorimetry (DSC) under a nitrogen atmosphere at a heating rate of 10 °C min^−1^. As presented in Fig. 2 and Table 1, these compounds were found to have excellent thermal stabilities with very high decomposition temperatures (*T*_d_, corresponding to 5% weight loss) of 375 °C and 452 °C for **SPy** and **DPy**, respectively. The glass transition temperature (*T*_g_) of **SPy** was 133 °C; however, the *T*_g_ of **DPy** was not detected. Such good thermal stability can be ascribed to their highly rigid structures and huge molecular weights, indicating their excellent film formation and making fabrication of OLED devices feasible through vacuum evaporation.

**Table 1:** Thermal, photophysical and energy properties of **SPy** and **DPy**

Emitter	*T*_g_*/T*_d_^a^/℃	*λ*_abs_^b^/nm	λ_em_^b/c^/nm	HOMO^d^/LUMO^e^/eV	*E*_S_/*E*_T_^f^/eV	PLQY^c^/%	FWHM^b^ /nm	*τ*_s_^g^ /ns
**SPy**	133/375	413	462/475	− 5.18/− 2.40	2.81/2.01	39.3	49	4.3
**DPy**	ND/452	443	472/483	− 5.10/− 2.43	2.72/1.94	66.5	37	3.9

^a^ *T*_d_ was measured by TGA (corresponding to 5% weight loss) and *T*_g_ was measured by DSC. ND stands for not detected. ^b^ Measured in dilute toluene at 298K. ^c^ Absolute fluorescence quantum yield obtained in PMMA films (1 wt%). ^d^ HOMO was estimated from the CV. ^e^ LUMO = HOMO + *E*_opt_^g^. ^f^ Calculated from the onsets of fluorescence (FL, 77 K) and the phosphorescence (Phos, 77 K) spectra. ^g^ Measured in PMMA films (doping consentration: 1 wt%)

**Fig. 3:**
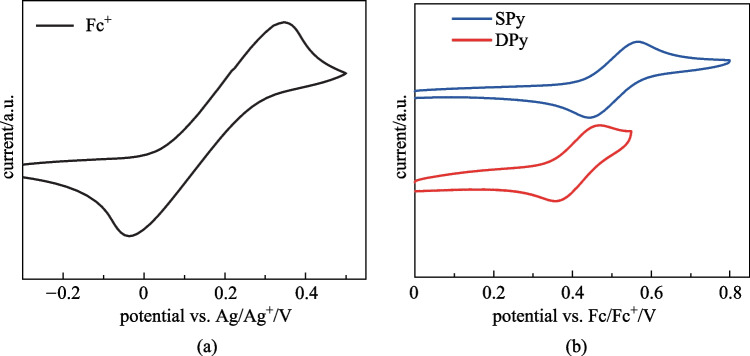
CV curves of **a** ferrocene (Fc) and **b** **SPy** and **Dpy**

The electrochemical properties were investigated by cyclic voltammetry (CV) measurement using a traditional three-electrode system to better understand the HOMO/LUMO of these compounds. As shown in Fig. 3, from the onsets of oxidation potentials, the HOMO energy levels of **SPy** and **DPy** were calculated to be − 5.18 and − 5.10 eV, respectively. Based on the HOMO values and the optical gap (*E*_opt_^g^) obtained from the absorption spectrum, the LUMO energy levels were estimated to be − 2.40 and − 2.43 eV, respectively (Table 1), using the equation *E*_LUMO_ = *E*_HOMO_ + *E*_opt_^g^.

### Photophysical properties

**Fig. 4:**
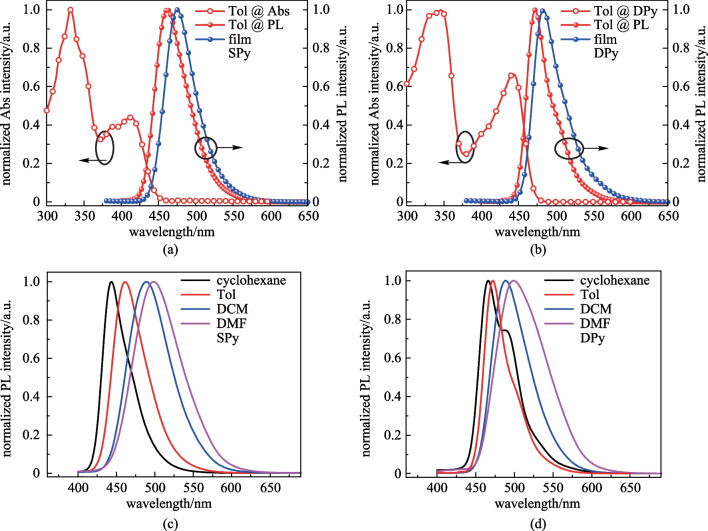
UV–vis absorption and PL measurement results. **a**, **b** are UV–vis absorption (Abs, room temperature) and fluorescence (Fl, room temperature) in dilute toluene (Tol), as well as fluorescence (Fl, room temperature) in film. **c**, **d** are fluorescence (Fl, room temperature) spectra in different solvents (cyclohexane, Tol, dichloromethane (DCM), and *N*,*N*-dimethylformamide (DMF)). **a**, **c** are for **SPy**, **b**, **d** are for **DPy**

The ultraviolet–visible (UV–vis) absorption, photoluminescence (PL) in dilute solutions and pure thin films of **SPy** and **DPy** were investigated. The results are shown in Fig. 4. Detailed photophysical parameters are listed in Table 1. The UV–vis absorption and PL were all measured at room temperature. For **SPy** and **DPy**, two prominent absorption bands were observed between 330 − 350 nm and 410 − 450 nm, and the absorption bands of DPy has a slight red-shift relative to that of SPy. This was in agreement with previous theoretical calculation. The optical band gaps were estimated to be 2.78 and 2.67 eV from the absorption onset. The emission peaks of **SPy** and **DPy** in diluted toluene solutions were located at 462 and 472 nm with small FWHM of 49 and 37 nm, respectively. As the geometrical relaxation between the ground state and the excited state can be restrained due to the introduction of 9,9-diphenyl-*N*-phenyl-9H-fluoren-2-amine bilaterally, the Stokes shift of **DPy** was as small as 29 nm. In addition, the films exhibited a bathochromic shift of the emission peak of 13 nm for **SPy** and 11 nm for **DPy**, which could be attributed to the enhanced intermolecular π-π interaction [30]. Figures 4c, d show the PL emission of **SPy** and **DPy** in different solvents at room temperature. The fluorescence of **DPy** showed a smaller red-shift than that of **SPy** with the increase of the solvent polarity. **SPy** and **DPy** exhibited a total red-shift of 56 and 32 nm, respectively, with environmental polarity increasing from nonpolar cyclohexane to the most polar *N*,*N*-dimethylformamide. Notably, Such a small red-shift of **DPy** (32 nm) demonstrated a weak intramolecular charge transfer (ICT) in the excited state, restraining the dissipation of energy. The emission properties were strongly affected by the nature of singlet excited states (S_1_) and the polarity of the local environment, as seen from the PL spectra in different solvents. Emission spectral profiles of **DPy** presented fine vibration features of locally excited (LE) states in low-polarity solvent of cyclohexane, and the emission spectral profiles evolved into Gaussian-type band shape gradually with the increase of the polarity of solvent. The emission spectral profiles of **DPy** in toluene were derived from the transition of Gaussian-type band shape of charge transfer (CT) states, featuring a narrower emission band than that in cyclohexane.

**Fig. 5:**
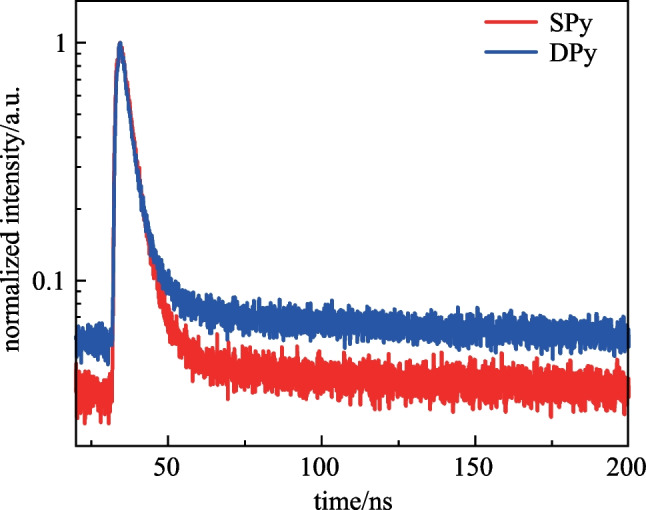
Transient PL spectra of **SPy** and **DPy** doped into PMMA films

The low-temperature fluorescence (Fl) and phosphorescence (Phos) spectra of the two compounds at 77 K in toluene are shown in Additional file 1: Fig. S1. The Phos emission peaks of these compounds were 642 and 670 nm for SPy and DPy, respectively. The lowest singlet (S_1_)/lowest triplet state (T_1_) energies estimated from the onsets of the emission spectra were calculated to be 2.81/2.01 eV and 2.72/1.94 eV for SPy and DPy, respectively. Furthermore, the low-temperature fluorescence spectrum of **SPy** exhibited a sharp narrowband emission with a small FWHM of 37 nm, which presents a striking difference from that of PL emission at room temperature, demonstrating the geometrical relaxation between the ground state and the excited state [34]. The absolute photoluminescence quantum yield (PLQY) in PMMA films obtained using the calibrated integrating sphere system were 39.3% for **SPy** and 66.5% for **DPy**. As depicted in Fig. 5, the transient PL decay curves of the oxygen-free doped film (1 wt%-doped in PMMA) were measured. **SPy** and **DPy** merely showed single-exponential attenuation in a nanosecond time scale with lifetimes (*τ*_s_) of 4.3 and 3.9 ns, respectively. This demonstrated the emission only originated from transient radiation of S_1_ state and was not TADF type emission, which was consistent with the theoretical calculation.

### OLED device performance

To evaluate the EL performance of **SPy** and **DPy**, the optimized non-doped OLEDs devices were firstly fabricated with the architecture of indium tin oxide (ITO) /MoO_3_ (10 nm)/TAPC (60 nm)/mCP (10 nm)/emitter (20 nm)/DPEPO (5 nm)/TmPyPB (40 nm)/LiF (1 nm)/Al (100 nm), and **SPy** for Device A, **DPy** for Device C. The energy level diagram and related molecular structures are shown in Additional file 1: Fig. S2, where 1,1-bis[(di-4-tolylamino)phenyl] cyclohexane (TAPC), 5-tri(mpyrid-3-yl-phenyl)benzene (TmPyPB) and *m*CP, bis[2-(diphenylphosphino)phenyl] ether oxide (DPEPO) were used as the hole transporting layer, the electron transporting layer and electron blocking layer, respectively.

**Fig. 6:**
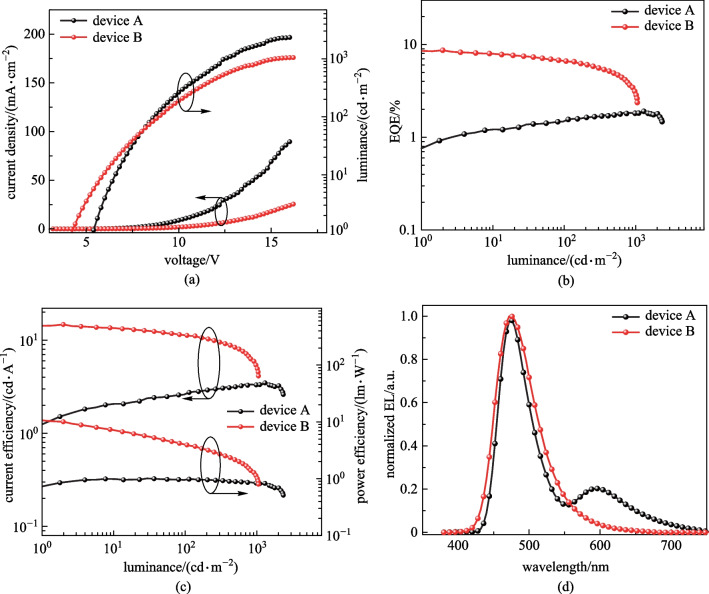
**a** *J-V-L* curves; **b** EQE versus luminance curves; **c** CE-*L*-PE curves and **d** normalized EL spectrum at 6 V. (In devices A and B, the EMLs of **SPy** and DPEPO: DMAC-DPS: **SPy** are used, respectively)

**Fig. 7:**
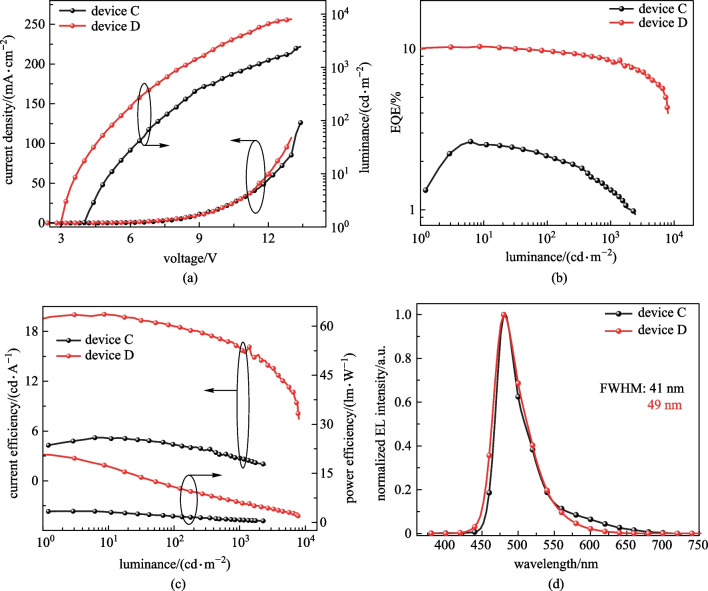
**a** *J-V-L* curves; **b** EQE versus luminance curves; **c** CE-*L*-PE curves and **d** normalized EL spectrum at 6 V. (In devices C and D, EMLs of **DPy** and DPEPO:DMAC-DPS:**DPy are used**, respectively)

The EL spectrum and other device characteristics related to efficiencies, luminance (*L*), voltages (*V*), and current densities (*J*) are presented in Fig. 6 for the devices based on **SPy** and in Fig. 7 for the devices based on **DPy.** Detailed device parameters are summarized in Table 2. These results illustrated that the maximum EQEs of devices A and C were 1.9% and 2.6%, respectively. **Spy-** and **DPy**-based OLEDs exhibited sky-blue emission with peaks at 472 and 480 nm, respectively. In addition, the EL spectrum of the device A exhibited a new peak, where the red-shifted band centered at 600 nm appears to have arisen from the exciplex emission. This can be confirmed with the EL spectrum at different voltages as shown in Additional file 1: Fig. S4. The exciplex emission is enhanced with increasing voltage [47]. In addition, the phenomenon in device C is alleviated, demonstrating the symmetrical molecule structure of DPy has a certain inhibitory effect on concentration quenching.

**Table 2:** EL Performance of the **SPy** and **DPy**-based devices

Device	*V*_on_^a^/V	*L*_max_^b^/(cd⋅m^−2^)	CE_max_^c^/(cd⋅A^−1^)	PE_max_^d^(lm⋅W^−1^)	EQE^e^/%	*λ*_EL_^f^/FWHM^f^/nm	CIE^g^(*x*, *y*)
A	5.4	2342	3.5/2.8/3.3	0.8/1.0/0.8	1.9/1.5/1.8	472/50	(0.23, 0.27)
B	4.2	1053	14.7/11.3/5.3	10.1/3.9/1.1	8.7/6.6/3.0	472/65	(0.15, 0.24)
C	4.0	2436	5.2/4.4/2.7	3.4/1.9/0.8	2.6/2.2/1.3	480/41	(0.17, 0.36)
D	3.0	7997	20.1/18.6/16.0	17.5/10.8/6.1	10.4/9.7/8.5	480/49	(0.14, 0.32)

^a^Turn on voltage at 1 cd/m^2^. ^b^Luminance: maximum values at 100, 1000 cd/m^2^. ^c^Current efficiency: maximum values at 100, 1000 cd/m^2^. ^d^Power efficiency: maximum values at 100, 1000 cd/m^2^. ^e^External quantum efficiency (%): maximum values at 100, 1000 cd/m^2^. ^f^The peak of EL spectrum and the full width at half maximum of EL spectrum. ^g^Commission Internationale de l’Eclairage coordinates. EML: A (SPy), B (DPEPO:DMAc-DPS:SPy), C (DPy), D (DPEPO:DMAc-DPS:DPy)

**Fig. 8:**
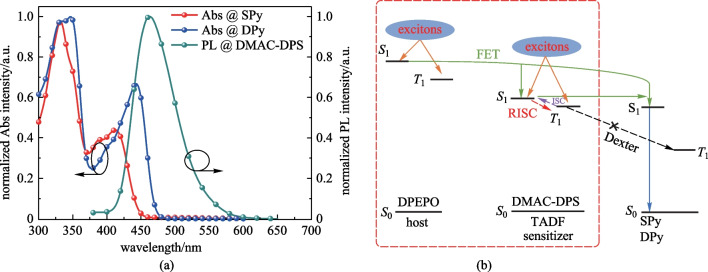
**a** UV–vis absorption spectrum of **SPy**, **DPy** and PL spectra of DMAC-DPS and **b** energy transfer process for device B and D

To further improve the performance of our emitters, highly efficient TSF devices are proposed. The well-known TADF material DMAC-DPS was chosen because of the highly efficient energy transfer between DMAC-DPS and our emitters (shown in Fig. 8) [48]. In order to obtain better energy transfer, the emitting material layer (EML) composed of DPEPO: DMAC-DPS: emitters were designed. The TSF device was constructed in the following order: ITO /MoO_3_ (10 nm)/TAPC (50 nm)/mCP (10 nm)/DPEPO: 40 wt% DMAC-DPS: 1 wt% emitters (20 nm)/ TmPyPB (40 nm)/LiF (1 nm)/Al (100 nm), **SPy** for Device B, **DPy** for Device D. The energy diagram of the device and structure of materials are shown in Additional file 1: Fig. S3. Herein, bis[2- (diphenylphosphino)phenyl]ether oxide (DPEPO) (*T*_1_ = 3.3 eV) acted as host. Predictably, the maximum EQE of DMAC-DPS TSF devices were 8.7% for device B and 10.4% for device D. Device D based on **DPy** exhibited higher EQE owing to its larger spectral overlap and higher PLQY. Although both TSF devices had broader spectra than those of fluorescent devices influenced by high host polarity, the FWHM of device D maintained a satisfactory level (49 nm). The maximum luminance (*L*_max_), current efficiency (CE_max_) and power efficiency (PE_max_) of device D were 7997 cd/m^2^, 20.1 cd/A, and 17.5 lm/W, respectively. From the non-doped OLEDs devices to the TSF devices, *L*_max_ of **DPy** was improved, while the *L*_max_ of **SPy** was reduced. In a TSF system, the absorption spectrum of fluorescent dopant overlaps well with the emission spectrum of TADF-sensitizer, thus more efficient energy transfer can be anticipated. Figure 8a shows that the absorption spectrum of **SPy** dopant overlaps to a small extent with the emission spectrum of DMAC-DPS, resulting in an inadequate energy transfer. Therefore, *L*_max_ of **SPy** was reduced from the non-doped OLEDs devices to the TSF devices. The turn-on voltages of the three types devices C and D were 4.0 and 3.0 V, respectively. The complicated EML system was well matched with the adjacent layer in terms of HOMO/LUMO levels, which demonstrated the smallest energy barrier favour of easy injection of electrons and holes. The blue index was calculated with the values of 61.0 and 62.8 for Device B and D, respectively. The higher blue index and narrowband emission for **DPy**-based TSF device was in accordance with the adequate energy transfer between the TADF-sensitizer and the emitter of **DPy**. More impressively, the efficiency roll-off characteristics were considerably improved. These results demonstrated that TADF-sensitized narrowband fluorescence is important for the improvement of fluorescence device performance.

## Conclusions

In summary, two new narrowband fluorescent emitters consisting of a pyrene unit, namely **SPy** and **DPy** were designed and synthesized. Accordingly, **DPy** exhibited narrowband blue emission with* λ*_em_ of 472 nm and a small FWHM of 37 nm. It also exhibited a small Stokes shift of 29 nm demonstrating that the geometrical relaxation between the ground state and the excited state can be restrained due to the introduction of 9,9-diphenyl-*N*-phenyl-9H-fluoren-2-amine bilaterally. Consequently, with an efficient spectral overlap between **DPy** and DMAC-DPS, highly efficient OLEDs were obtained by a sensitization strategy. The blue fluorescent OLEDs utilizing **SPy** and **DPy** as emitters achieved a maximum EQEs of 8.7% and 10.4%, respectively, with the EL peak of 472 and 480 nm, respectively. In particular, the EQE of **DPy**-based device was boosted from 2.6% in non-doped device to 10.4% after using DMAc-DPS as sensitizer. We believe that our molecular design strategy will be benefical for the exploration of highly efficient fluorescent emitters.

## Supplementary Information

Below is the link to the electronic supplementary material.

Additional file 1. Additional figures and sections.
