# Supplementary material for: A sensitization strategy for highly efficient blue fluorescent organic light-emitting diodes
Source: Front Optoelectron. 2022 Nov 10;15(1):44. doi: 10.1007/s12200-022-00046-z (PMC9756245; doi:10.1007/s12200-022-00046-z)
Supplement: Supplementary file 1 — Additional file 1. Additional figures and sections. [file 12200_2022_46_MOESM1_ESM.docx]

**Additional file 1**

**Sensitization strategy for** **highly** **efficient** **blue fluorescent organic light-emitting diodes**

Yalei DUAN, Runda GUO, Yaxiong WANG, Kaiyuan DI, Lei WANG (🖂)

Wuhan National Laboratory for Optoelectronics, Huazhong University of Science and Technology, Wuhan, 430074, China

Email: [wanglei@mail.hust.edu.cn](mailto:wanglei@mail.hust.edu.cn)

**Characterization.**

The intermediates and final compounds were confirmed by 1H NMR or 13C NMR spectra on a Bruker-AF301 AT 400 or 600 MHz spectrometer. Mass spectra were recorded on high resolution atmospheric chemical ionization (APCI) mass spectrometer.

Differential scanning calorimetry (DSC) was measured under nitrogen on a PE Instruments DSC2920 unit at a heating rate of 10 °C min^-1^ from 30 to 300 °C. The glass transition temperature (T_g_) was determined from the second heating scan. Thermogravimetric analysis (TGA) was undertaken using a PerkinElmer Instruments Pyris1 TGA at a heating rate of 10 °C min^-1^ from 30 to 600 °C under a nitrogen atmosphere. The thermal decomposition temperatures (T_d_) were corresponded to 5% weight loss temperatures.

The UV-vis absorption spectra were measured on a Shimadzu UV-VIS-NIR Spectrophotometer (UV-3600) in the wavelength range of 190 ~ 1100 nm and the PL spectra were recorded on an Edinburgh Instruments (FLS 920 spectrometer). The transient photoluminescence spectra were recorded modular fluorescence spectrometer (HORIBA Corporation of Canada, QuantaMaster 8000). The absolute PLQYs were estimated on a Quantaurus QY measurement system (C11347-11, Hamamatsu Photonics).

Cyclic voltammetry was recorded on a computer-controlled EG&G Potentiostat/Galvanostat model 283 at room temperature with a conventional three-electrode system, which consisted of a platinum wire counter electrode, an Ag/AgNO_3_ (0.1 M) reference electrode, and a glassy carbon working electrode of 2 mm diameter. A 0.10 M tetrabutylammonium hexafluorophosphate (*n*-Bu_4_NPF_6_) solutions in dry dichloromethane and *N*, *N*-dimethylformamide were employed as the supporting electrolyte, and ferrocene was added as a calibrant in the whole measurement.

Device fabrication and measurements.

**Calculation details.**

The density functional theory (DFT) and timed dependent DFT (TD-DFT) calculations were employed to optimize the ground state and excited state geometries and electronic properties, which were carried out with the B3LYP hybrid functional at the basis set level of 6-31G(d).

**Device fabrication and measurements.**

The used ITO glass substrates, MoO3, LiF, di-(4-(N, N-ditolylamino)phenyl)cyclohexane (TAPC) and 1,3-di(9H-carbazol-9-yl)benzene (mCP) were commercially available. The devices were fabricated by evaporating organic layers on ITO (20 Ω square^-1^), which were precleaned carefully and treated with oxygen plasma for 5 min. The devices were deposited in the vacuum of 2 × 10^-6^ Torr. For all of the OLEDs, the emitting areas were determined by the overlap of two electrodes as 0.09 cm^2^. The *J-V-L* of the devices was measured using a Keithley 2400 source meter equipped with a calibrated silicon photodiode. The EL spectra were measured using a PR655 spectrometer. All measurements were carried out at room temperature under ambient conditions.

**Fig. S1** Normalized fluorescence and phosphorescence spectra of (a) **SPy** and (b) **DPy** measured in toluene solution (77 K)

**Table S1** Summary of PL spectra data in different polar solvents of **SPy** and **DPy**

| **Emitter** | **SPy** | | **DPy** | |
| --- | --- | --- | --- | --- |
| **solvent** | **λ_em_^a^ (nm)** | **FWHM^b^ (nm)** | **λ_em_^a^ (nm)** | **FWHM^b^ (nm)** |
| cyclohexane | 444 | 41 | 466 | 51 |
| toluene | 462 | 49 | 472 | 37 |
| DCM | 489 | 64 | 489 | 55 |
| DMF | 498 | 72 | 499 | 76 |
| ^a^ PL peak wavelength. ^b^ Full width at half maximum | | | | |

**Fig. S2** The energy level diagram and molecular structures of the used materials in devices A and C

**Fig. S3** The energy level diagram and molecular structures of the used materials in devices B and D

Table S2. The EL performance of OLEDs based on Pyrene emitters

| emitters | λ_PL/EL_  (nm) | FWHM_PL/EL_ (nm) | EQE  (%) | Ref. |
| --- | --- | --- | --- | --- |
| **SPy** | **462/472** | **49/65** | **8.7** | **This work** |
| **DPy** | **472/480** | **37/49** | **10.4** | **This work** |
| 2CN | 454/456 | 46/40 | 4.0 | [1] |
| TP-AP-TP | 446/456 | 56/55 | 6.9 | [2] |
| 3 | 507/504 | -/46 | 2.0 | [3] |
| 3a | 393/396 | -/52 | 2.8 | [4] |

**Fig. S4** The EL spectrum of device A at different voltages


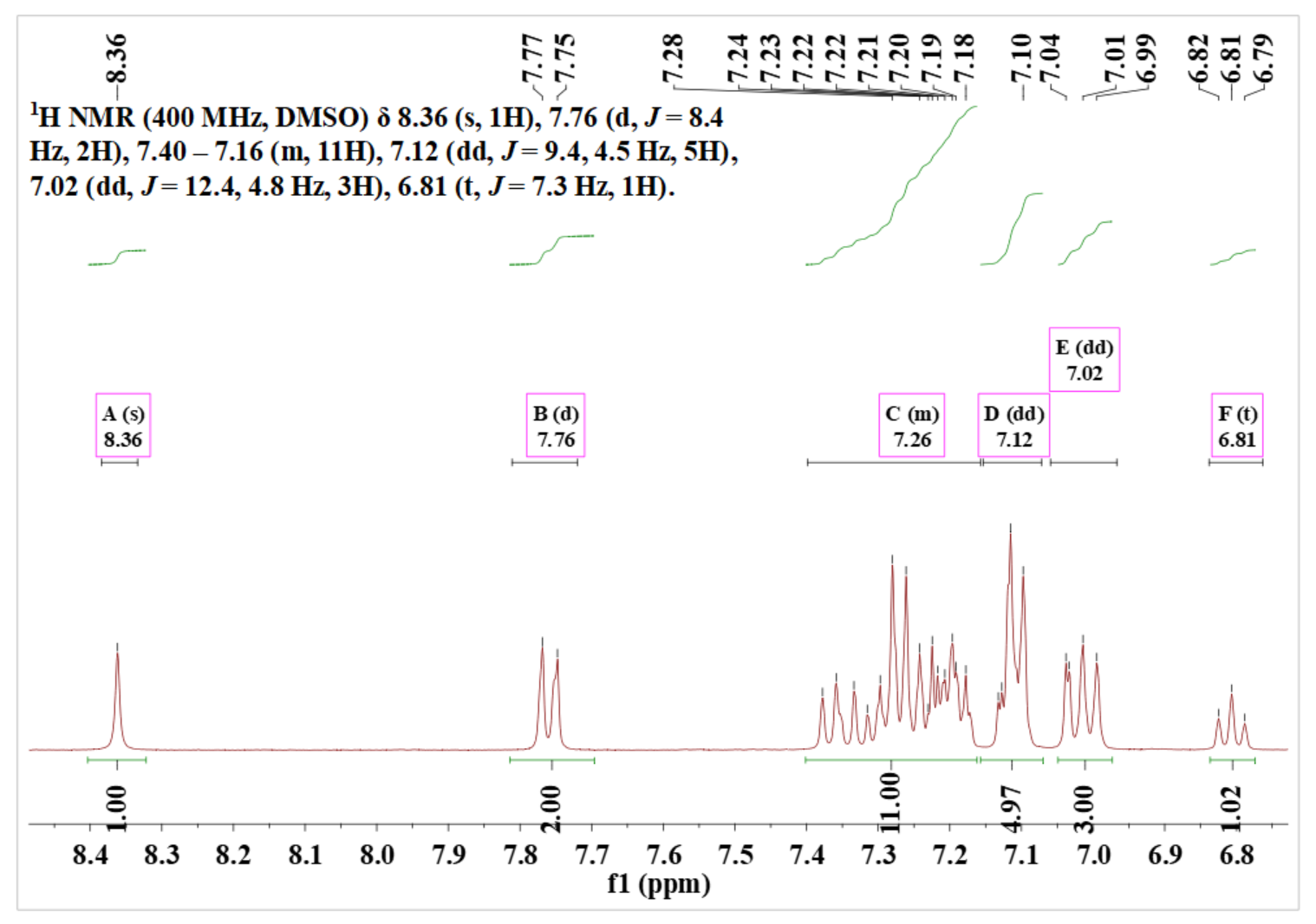

**Fig. S5** ^1^H NMR spectrum of **D1** (400 MHz, DMSO)


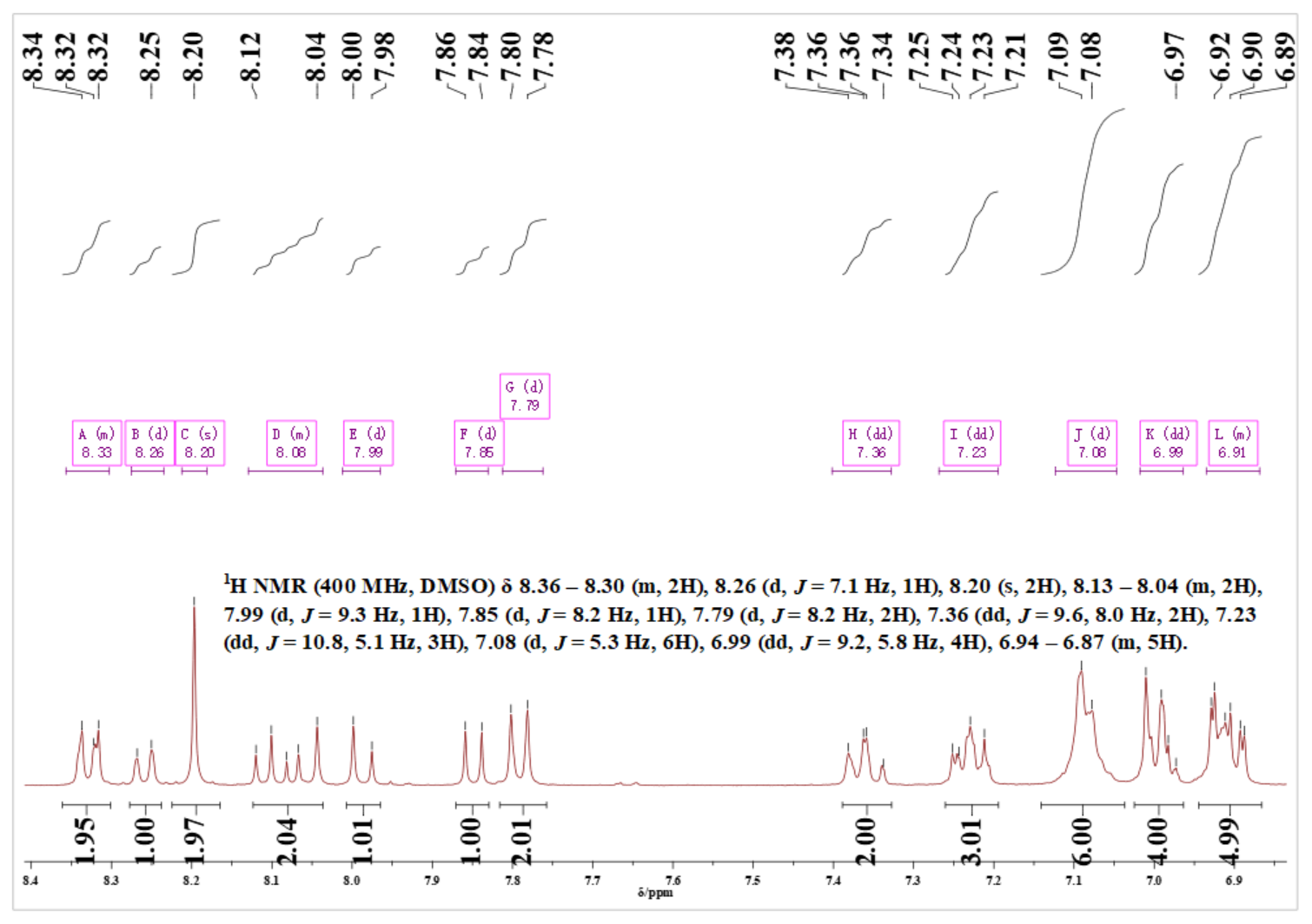

**Fig. S6** ^1^H NMR spectrum of **SPy** (400 MHz, DMSO)


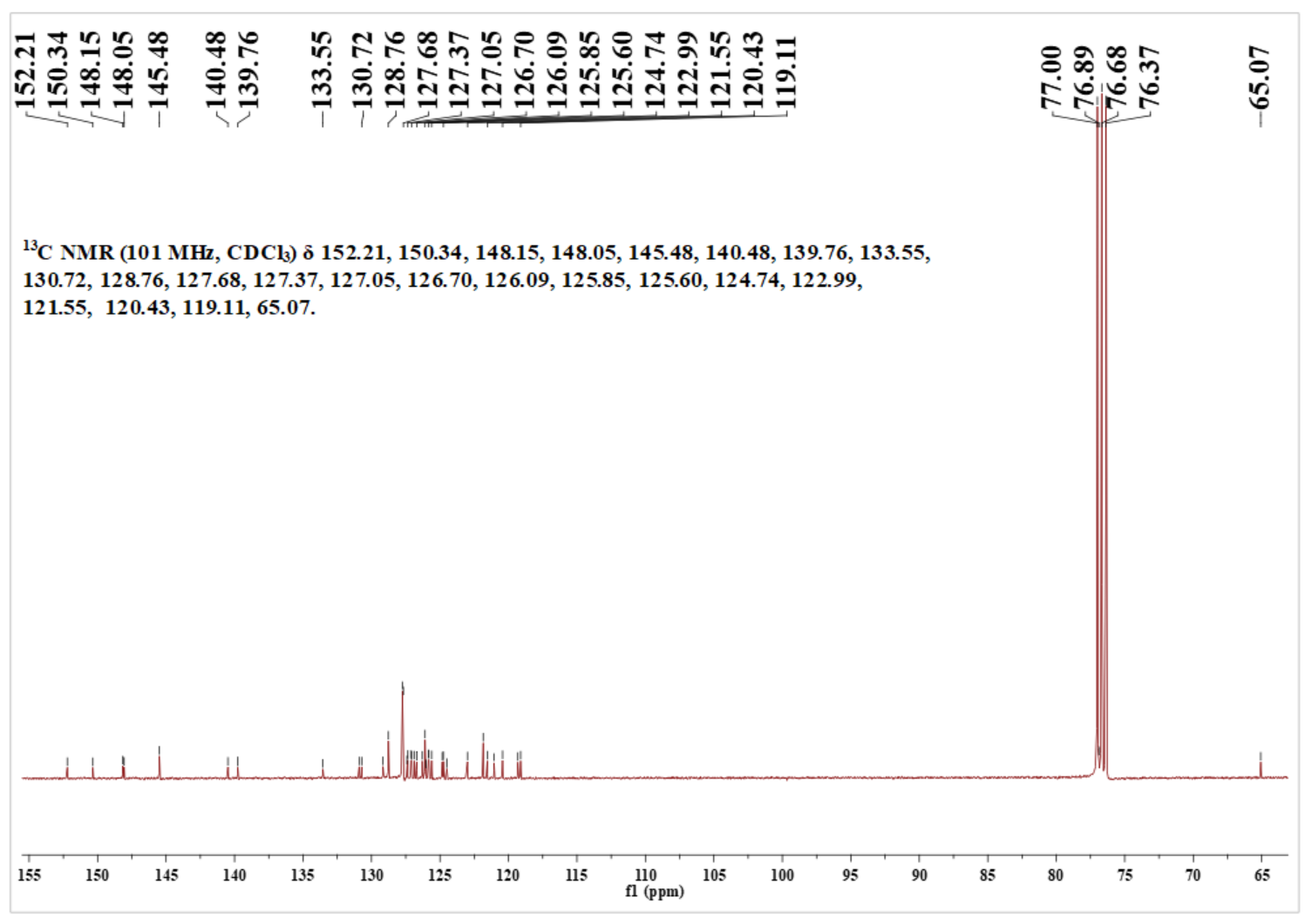

**Fig. S7** ^13^C NMR spectrum of **SPy** (101 MHz, CDCl_3_)


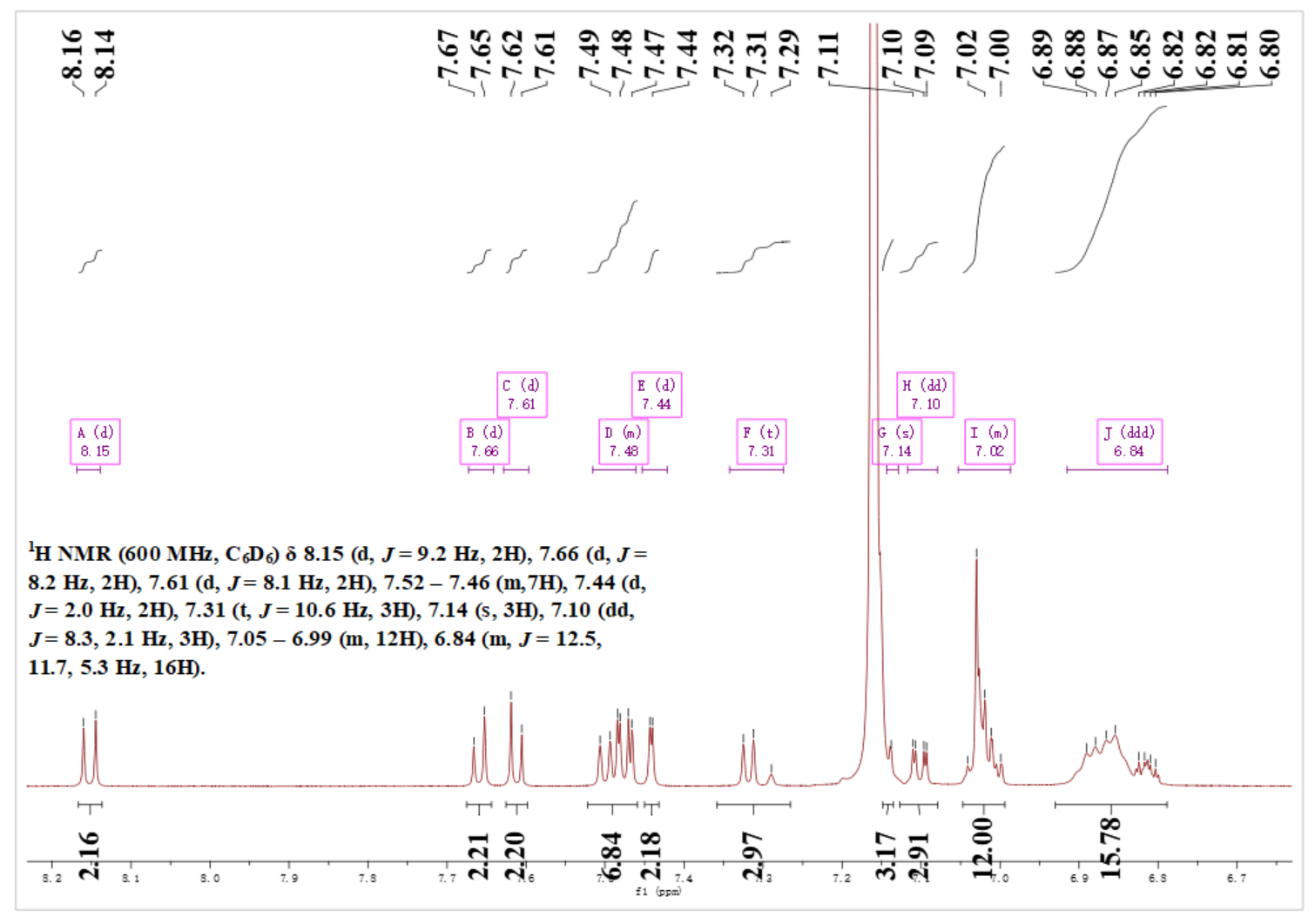

**Fig. S8** ^1^H NMR spectrum of **DPy** (600 MHz, C_6_D_6_)


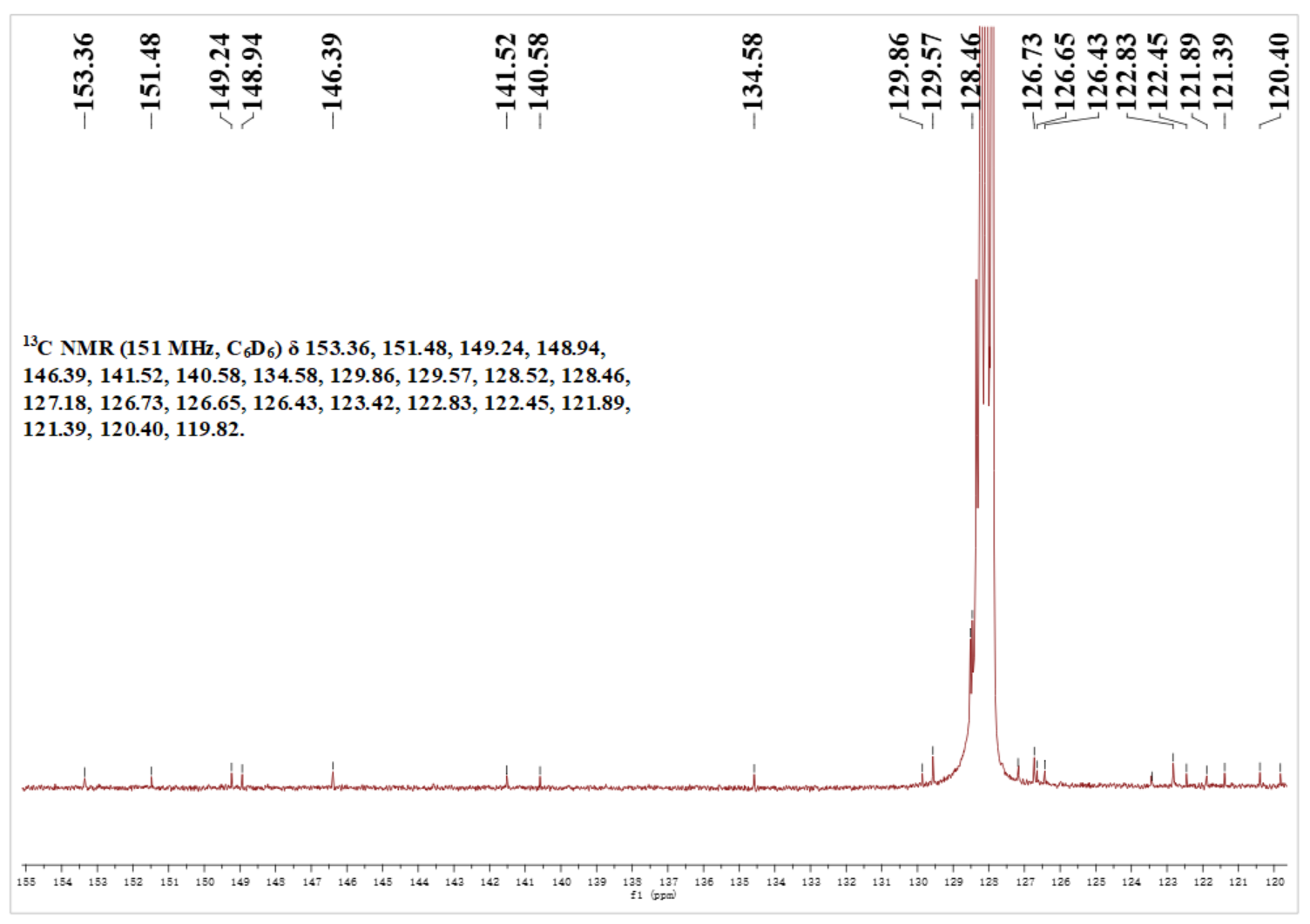

**Fig. S9** ^13^C NMR spectrum of **DPy** (151 MHz, C_6_D_6_)

# Reference

[1] K-R Wee, H-C Ahn, H-J Son, W-S Han, J-E Kim, D W Cho, et al. Emission color tuning and deep blue dopant materials based on 1,6-bis(n-phenyl-p-(r)phenylamino)pyrene. Journal of Organic Chemistry. 2009, 74: 8472-8475

[2] Y P Beomjin Kim, Jaehyun Lee, Daisuke Yokoyama, Ji-Hoon Lee, Junji Kidob and Jongwook Park. Synthesis and electroluminescence properties of highly efficient blue fluorescence emitters using dual core chromophores. Journal of Materials Chemistry C. 2013, 1: 432

[3] M-C H Rossatorn Muangpaisal, Tai-Hsiang Huang, Chih-Hsin Chen, Jiun-Yi Shen, Jen-Shyang Ni, Jiann T. Lin, Tung-Huei Ke, Li-Yin Chen, Chung-Chih Wue, Chiitang Tsai. Tetrasubstituted-pyrene derivatives for electroluminescent application. Organic Electronics. 2014, 15: 2148-2157

[4] V Joseph, K R J Thomas, S Sahoo, M Singh and J-H Jou. Asymmetrically 2,7-difunctionalized carbazole-based donor-acceptor hybrids for deep blue electroluminescence applications. Optical Materials. 2020, 108: 110159
